# Comparison of respondent-reported and sensor-recorded latrine utilization measures in rural Bangladesh: a cross-sectional study

**DOI:** 10.1093/trstmh/trx058

**Published:** 2017-11-20

**Authors:** Maryann G Delea, Corey L Nagel, Evan A Thomas, Amal K Halder, Nuhu Amin, Abul K Shoab, Matthew C Freeman, Leanne Unicomb, Thomas F Clasen

**Affiliations:** 1 Department of Environmental Health, Rollins School of Public Health, Emory University, Atlanta, Georgia, USA; 2 School of Public Health, Oregon Health and Science University, Portland State University, Portland, OR, USA; 3 Environmental Intervention Unit, International Centre for Diarrhoeal Disease Research, Bangladesh, Dhaka, Bangladesh

**Keywords:** Bangladesh, Public health, Sanitation, WASH

## Abstract

**Background:**

Health improvements realized through sanitation are likely achieved through high levels of facilities utilization by all household members. However, measurements of sanitation often rely on either the presence of latrines, which does not guarantee use, or respondent-reported utilization of sanitation facilities, which is prone to response bias. Overstatement of sanitation metrics limits the accuracy of program outcome measures, and has implications for the interpretation of related health impact data.

**Methods:**

We conducted a cross-sectional study of 213 households in 14 village water, sanitation and hygiene committee clusters throughout rural Bangladesh and used a combined data- and relationship-scale approach to assess agreement between respondent-reported latrine utilization and sensor-recorded measurement.

**Results:**

Four-day household-level respondent-reported defecation averaged 28 events (inter-quartile range [IQR] 20–40), while sensor-recorded defecation averaged 17 events (IQR 11–29). Comparative analyses suggest moderately high accuracy (bias correction factor=0.84), but imprecision in the data (broad scatter of data, Pearson’s *r*=0.35) and thus only weak concordance between measures (*ρ*_*c*_=0.29 [95% BCa CI 0.15 to 0.43]).

**Conclusions:**

Respondent-reported latrine utilization data should be interpreted with caution, as evidence suggests use is exaggerated. Coupling reported utilization data with objective measures of use may aid in the estimation of latrine use.

## Introduction

Globally, an estimated 2.4 billion people lack access to improved sanitation.^1^ Much of the sanitation challenge is focused in South Asia, where one-third of its 1.8 billion people still practice open defecation. In response, governments, nongovernmental organizations and others have undertaken large-scale programs to increase latrine coverage. Bangladesh has achieved success with such programs, as 95% of rural households have access to some form of sanitation facility.^2^ The country has observed progress over time, as the proportion of households (urban and rural) with an improved sanitation facility not shared with other households has increased from 34% in 2011 to 45% in 2014.^2^

International monitoring of sanitation relies principally on self-reported data. For example, the Joint Monitoring Programme on Water and Sanitation (JMP) recommends that national surveys assess sanitation by asking informants about the facilities that their household uses (JMP Core question 2006). While this may yield helpful estimates of sanitation access (coverage) and type of facilities, there is increasing evidence that it does not capture actual use. In neighboring India, for example, several studies have now shown that even amongst households with latrines, use is low or inconsistent, especially among men and children.^3–5^ Even these studies, however, may exaggerate actual latrine utilization.

Assessments of latrine use typically rely on respondent-reported practices, a measurement method that can be unreliable due to courtesy, social desirability and recall biases. Studies of respondent-reported handwashing, for example, have reported exaggerated compliance.^4,6–10^ Sensor-monitored use of cookstoves and water filters also suggests respondents tend to over-estimate environmental health behaviors that programs are designed to promote.^11^ Gaps between latrine coverage and actual use have also been reported in Ethiopia^12^ and Ghana.^13^ Deficiencies in use may at least partially explain why recent studies of sanitation interventions have failed to detect reductions in diseases such as diarrhea and soil-transmitted helminthiasis.^4,5,14,15^

In order to overcome potential bias associated with respondent reporting of latrine utilization, researchers developed the passive latrine use monitor (PLUM), a battery-operated device that can be discretely mounted inside latrines to detect use by recording motion.^16^ PLUMs have been used in India to determine methods of assessing latrine use that minimize bias.^17–19^ The objective of this research was to determine agreement between respondent-reported and PLUM-recorded latrine use and to ascertain whether reported latrine use data indicate evidence of bias relative to PLUM-recorded use.

## Methods

### Study context

This research was conducted within a cross-sectional verification study assessing the accuracy of implementer-reported sanitation outcomes from a water, sanitation and hygiene (WASH) program implemented throughout rural Bangladesh. The verification was a post-only study conducted in 52 village WASH committee (VWC) clusters from 177 program subdistricts (*upazilas*) in rural Bangladesh. Data were collected during June–August 2014, monsoon season in Bangladesh.

### Village and household selection

Data for this study were collected in 14 of the 52 verification VWC clusters. These clusters were eligible for inclusion in this latrine utilization study if they were included in the verification and had at least one household with a functional household latrine (i.e., a latrine that was working properly so that any household member could defecate and/or urinate at any given time with the necessary privacy [i.e., at least three walls and any type of door] and operability [i.e., not overflowing with feces]) with walls or a roof suitable for PLUM installation. A household was defined as a person or group of related or unrelated persons who usually live together in the same dwelling(s), have common cooking and eating arrangements and acknowledge one adult member as the head of household. All VWCs and households residing within the WASH program’s targeted subdistricts (*upazilas*) were eligible for inclusion in the verification study. Additional inclusion criteria required that households have at least one adult (i.e., an individual 18 years of age or older) who consented to participate in the verification study and serve as the primary survey respondent; this individual had to be capable of understanding and providing informed consent.

We employed a multilevel sampling strategy to randomly select VWCs and households for inclusion in the larger verification study, and subsequently this latrine utilization study. For the verification study, our sampling frame consisted of a line list of districts (*zilas*), subdistricts (*upazilas*), unions and VWCs targeted by the WASH program. We selected primary sampling units using probability proportional to size. During the next sampling stage, one VWC was selected from each union using simple random sampling. In the final sampling stage we identified potential study households by obtaining the household register from each selected VWC and stratifying the household sampling frame by wealth category, as defined by the VWC, to select eight households from each stratum. From the list of VWCs selected for the verification study, we used a random number generator to identify 14 VWCs in which we installed PLUMs in all functional and suitable latrines in each household selected for inclusion in the latrine utilization study.

### Data collection tools and methodologies

Enumerators collected data on latrine utilization using different measurement methods, including

1. Respondent-reported latrine use, collected via administration of a household latrine use schedule. This tool captured respondent- or self-reported latrine use for all regular latrine users, defined as members of the household or other individuals who are not members of the household but regularly use the household latrine(s) (e.g., neighbors, tenants, servants).
2. Sensor-recorded latrine utilization monitoring data captured via PLUM deployments in household latrines.

#### Respondent-reported latrine utilization data captured via structured household use schedule

In households randomly selected for inclusion in the study, enumerators sought out adult respondents, with a preference for the primary female caretaker of the youngest child within the household, who likely is most knowledgeable about the latrine use and defecation practices of most members of her household. Enumerators administered the household survey using electronic data capture on password-protected mobile devices that allowed for range and consistency checks. The survey instrument was designed to collect data on household demographics, wealth and socioeconomic status; latrine construction, maintenance and repair; latrine spot check indicators and reported latrine use and defecation practices. Within this survey instrument, enumerators administered a structured household latrine use schedule, which systematically captured reported data regarding latrine use and defecation practices for each regular user of the household’s latrine(s), including household members and other regular users. Enumerators asked all latrine users present during survey administration to provide self-reports of their own usual latrine utilization and defecation practices and asked the survey respondent to report for those who were not present during survey administration.

Each respondent was asked about his or her primary place of defecation, whether the primary place of defecation changes during the year and whether the latrine was always exclusively used for defecation. Enumerators subsequently asked about the number of times each household latrine user usually uses the latrine during four specific periods of the day—morning (i.e., 04:00–10:00), afternoon (i.e., 10:00–15:00), evening (i.e., 15:00–19:00), and night (i.e., 19:00–04:00).

#### Sensor-recorded latrine utilization data captured via PLUMs

After administering the survey, field staff installed one PLUM device in each functional and suitable (see above-mentioned definitions) household latrine in all consenting selected households. We used a fourth-generation PLUM device developed by co-authors from Portland State University (http://www.pdx.edu/sweetlab). Details regarding earlier generations of similar devices, field testing, validation and the algorithm are described elsewhere.^16^ PLUMs collected electronic sensing data in the latrines over the course of a 1-week installation period.

### Assessing agreement between latrine utilization measurement methods

We conducted an analysis of household-level paired data (i.e., reported latrine use data paired with PLUM-recorded use data) to compare latrine utilization metrics from the subset of households with successful PLUM installations. To assess agreement between latrine utilization measurements, we quantified latrine use count data generated from the respective measurement methods, as indicated below. For both measures, latrine utilization was calculated via the quantification of 4-day, household-level ‘likely defecation’ events. For the PLUM-recorded metric, we define the 4-day, household-level ‘likely defecation’ event total as a sum of all events recorded during days 3–6 of the PLUM installation period (i.e., a 4-day PLUM analytical period). In order to minimize reactivity observed during the first days of a PLUM installation period (e.g., curious children and adults entering the latrine to look at the sensor, people potentially modifying their latrine use behaviors), we dropped the first 2 days of PLUM data, analyzing data from these 4 days. For respondent-reported events, we defined this 4-day, household-level metric by summing the reported individual-level ‘usual’ latrine use events across the four specific periods of the day and then summing the individual-level totals for all latrine users older than 3 years of age. In order to generate data that were comparable to a 4-day PLUM-recorded measure, we multiplied the household-level ‘usual’ event total by 4.

#### Respondent-reported latrine use measures

Respondent-reported use measures reflect a 4-day, household-level reported latrine use total for all household latrine users over the age of 3 years. Reported use for children under 3 years of age was excluded from the household-level total number of reported daily ‘likely defecation’ events because the PLUM algorithm has not been validated to capture child feces disposal and latrine training events, and inclusion of these events in the reported use total could threaten the comparability of utilization measurements between the two methods.

#### PLUM-recorded latrine use measures

PLUM-recorded use measures reflect a 4-day, household-level, sensor-detected latrine use total. We generated these data by employing a validated algorithm to assess raw PLUM signal data and detect and quantify signal patterns indicative of ‘likely defecation’ events.

During the data collection period we experienced several technical issues with the PLUM sensors that resulted in data loss. Any household meeting one or more of the following criteria were dropped from the analytical PLUM sample:

1. Households with a malfunctioning PLUM that registered faulty data on one or more days;
2. Households with a malfunctioning PLUM in one household latrine but functioning PLUMs in the remaining household latrines. These households were dropped from the analytical sample because data loss from one or more PLUM sensors would prevent us from generating an accurate PLUM-recorded use measure for those respective households; and
3. Households that accepted PLUM installation in one latrine but refused PLUM installation in one or more subsequent latrines. These households were also dropped from the analytical sample because we would be unable to generate an accurate PLUM-recorded use measure for these respective households as well.

We analyzed data in Stata 13.1 (StataCorp, College Station, TX, USA), using user-written *batplot* and *concord* packages.^17^ To assess agreement between respondent-reported and PLUM-recorded methodologies, we combined the data-scale approach recommended by Bland and Altman’s limits of agreement (LoA)^18^ with the complementary relationship-scale approach suggested by Lin’s concordance correlation coefficient (CCC), a scaled index of agreement.^19,20^ The magnitude of the CCC reflects both the precision and accuracy of the data and can be used to determine whether the observed data deviate from the line of perfect concordance. This method allowed for an analysis of the accuracy and precision of the data that is not provided by the limits of agreement approach in the absence of a scaled index of agreement. We performed a model II, least-products regression to fit a reduced major axis to the data in a concordance plot. We employed a bootstrap resampling technique (1000 replications) to generate a bias-corrected and accelerated bootstrap confidence interval (BC_a_) for the CCC presented herein.^21^ Details related to the steps executed to generate relevant statistics and produce modified Bland–Altman and concordance plots are outlined in the supplementary material.

### Data structure and assumptions

Arguably, no ‘gold standard’ latrine utilization measurement method exists; therefore, we used a data structure that consisted of discrete utilization measures, without replications, and no assumed ‘gold standard’ measurement method.

## Results

### Sample household characteristics

**Figure 1.**
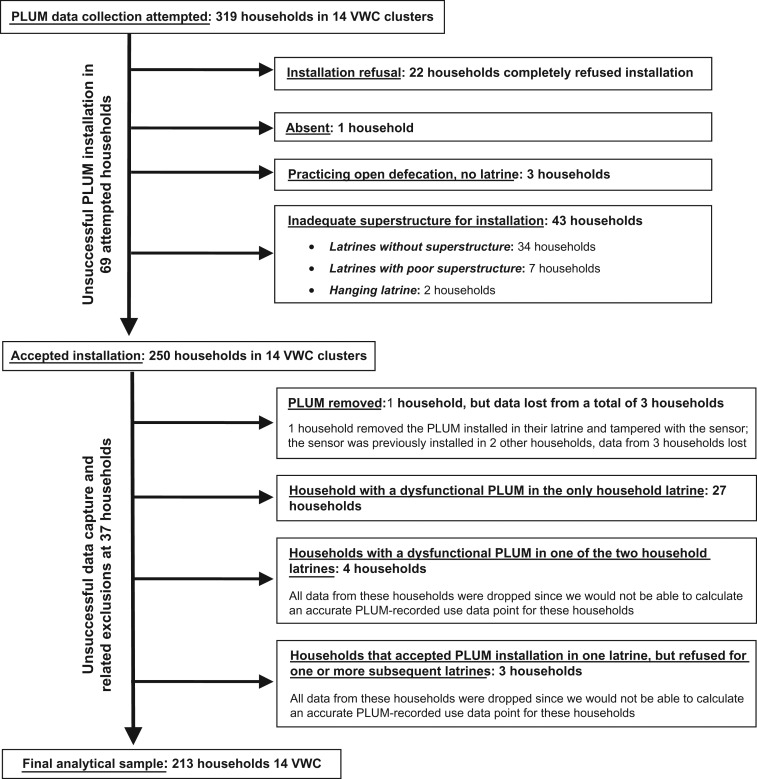
Flow of PLUM data capture.

The field team was given consent to install PLUMs in 250 of 319 households targeted for PLUM installation, resulting in an installation success rate of 78%. The final analytical sample included data from 213 households in the 14 VWC clusters, from which respondent-reported demographic and utilization data were captured along with PLUM-recorded utilization data. This translated to an 85% (213/250) data extraction rate among successful installations. Table 1 summarizes sample household characteristics. Figure 1 outlines the flow of PLUM data capture and indicates reasons for household-level exclusion.

**Table 1.** Sample household characteristics

Characteristic	n (%)
Total number of functional household latrines	
One functional household latrine	198 (93.0)
Two functional household latrines	14 (6.5)
Three functional household latrines	1 (0.5)
Type of household latrine	
One or more functional, improved and not shared	120 (56.3)
One or more functional, shared but otherwise improved*	30 (14.1)
One or more functional unimproved only†	63 (29.6)
Household wealth category‡	
Nonpoor	79 (37.1)
Poor	62 (29.1)
Ultra-poor	72 (33.8)
Highest educational attainment of the head of household	
No formal education	81 (38.0)
Some primary schooling	30 (14.1)
Completed primary schooling	27 (12.7)
Some secondary schooling	54 (25.3)
Completed secondary schooling or higher	21 (9.9)
Total number of households	213
**Characteristic**	**Median (IQR)**
Total users of household latrine	5 (4–7)
Total household members	5 (4–6)
Proportion household latrine users self-reporting defecation practices	25.0% (16.7–40.0)

We employed JMP definitions to distinguish improved and unimproved sanitation facilities. We also present data on ‘shared, but otherwise improved’ latrines separately. See ‘Methods’ section for details regarding the operational definition of ‘functional latrine’.

* One or more functional, shared but otherwise improved latrine; no access to an improved household latrine.

† One or more functional unimproved latrine with no access to a functional improved or shared but otherwise improved household latrine.

‡ Household wealth category, per implementing nongovernmental organization’s 2007/2012 census, as indicated in VWC registers.

Data on defecation and latrine utilization practices were captured from a total of 1363 household latrine members from the 213 latrine utilization study households. Of those latrine users, 27% (363/1363 individuals) provided self-reported data on latrine use and defecation practices; the remaining data were provided via proxy. The average household-level proportion of self-reporting latrine users was similar (25% [interquartile range {IQR} 17–40]).

### Respondent-reported and PLUM-recorded utilization distributions

The 4-day, household-level, respondent-reported ‘likely defecation’ events averaged 28 events (IQR 20–40) and the number of PLUM-recorded ‘likely defecation’ events averaged 17 events (IQR 11–29). Given that the median number of household latrine users was 5 (IQR 4–7), this translates to 1.4 respondent-reported ‘likely defecation’ events per person per day and 0.85 PLUM-recorded ‘likely defecation’ events per person per day. Utilization data generated by the two measurement methods were found to have similar distributions (Figure S1), meaning the data did not require further adjustment prior to conducting comparative analyses.

### Tests of assumptions

See the supplementary material for details regarding results generated from the tests of assumptions. As neither the bias nor the variance were constant over the range of utilization values (i.e., violating assumptions of the traditional Bland–Altman limits of agreement approach), it was necessary to employ a modified Bland–Altman approach that adjusted for nonconstant variance and bias.

### Assessment of limits of agreement

**Figure 2.**
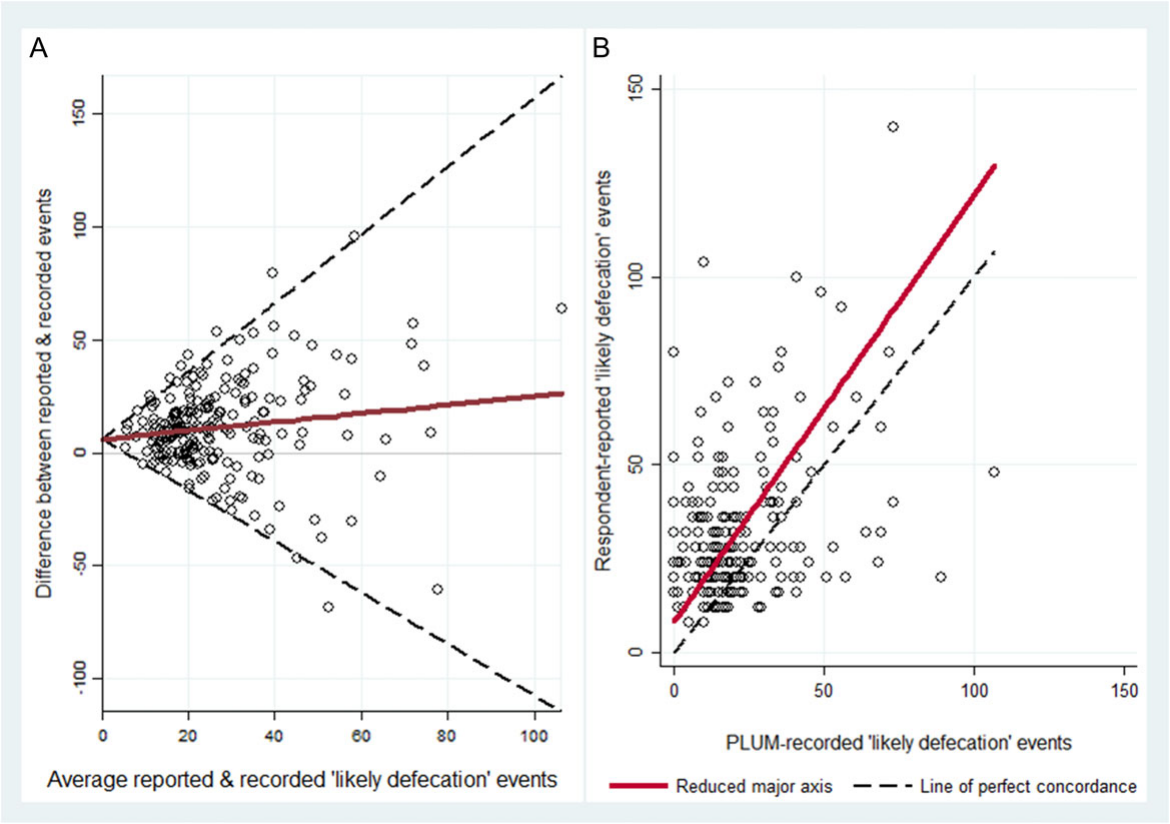
(Panel A) The modified Bland–Altman plot suggests overreporting of respondent-reported likely defecation latrine events, as indicated by the upward bias (i.e., upward slope) of the mean measurement line (i.e., the solid bold line) relative to zero. The relatively wide 95% LoA indicates poor agreement between respondent-reported and PLUM-recorded latrine utilization measures. (Panel B) The concordance correlation plot indicates bias in respondent-reported latrine utilization, as indicated by the solid reduced major axis falling above the dashed line of perfect concordance. The reduced major axis falls moderately close to the line of perfect concordance and, along with the bias correction factor (0.84), indicates moderately high accuracy in the latrine use measures. However, the broad scatter of the observations from the reduced major axis and the magnitude of Pearson’s *r* (0.35) suggest imprecision in the data. As such, evidence suggests only a weak, yet significant concordance correlation between reported and recorded latrine utilization measures (*ρ*_*c*_=0.29 [95% BCa CI 0.15 to 0.43]).

When we assessed the difference between and average of latrine utilization measurement methods, we found an upward bias in the difference between measurements relative to the average, indicating overestimation of respondent-reported utilization relative to PLUM-recorded utilization. The modified Bland–Altman plot indicated a relatively wide 95% LoA, which suggests poor agreement between the two latrine utilization measures from the data-scale perspective (Figure 2, panel A).

### Assessment of indices of agreement

The slope of the reduced major axis was found to be 1.14, which reflects the sign of Pearson’s *r* and the ratio of the standard deviations (Figure 2, panel B). The intercept of the reduced major axis was 8.2. These statistics indicate that respondent-reported use was, on average, upwardly biased (i.e., overreported) and the magnitude of bias (i.e., amount of overreporting) of respondent-reported latrine utilization increases as the number of events increases, as evidenced by the gradient of the relationship between the two methods exceeding 1.0.

The CCC between respondent-reported and PLUM-recorded utilization was *ρ*_c_=0.29 (95% BCa CI 0.15 to 0.43). This CCC indicates that respondent-reported ‘likely defecation’ events are only weakly correlated with PLUM-recorded ‘likely defecation’ events. The reduced major axis was moderately close to the line of perfect concordance, and the bias-correction factor was 0.84, which indicates a moderately high level of accuracy in the measures. However, the data were imprecise, as indicated by Pearson’s *r*=0.35, and the broad spread of observations from the reduced major axis. These findings help explain the relatively weak concordance correlation between reported and recorded latrine utilization measures.

## Discussion

Findings from both data- and relationship-scale agreement assessments indicate an upward bias in respondent-reported latrine utilization measures, suggesting overreporting of latrine use relative to PLUM-recorded use. These findings are demonstrated through the upward slope of the mean measurement line relative to zero in Figure 2, panel A, and the positioning of the reduced major axis above the line of perfect concordance in Figure 2, panel B. Results from our data-scale LoA approach suggest a relatively poor magnitude of agreement between respondent-reported and PLUM-recorded latrine use measures, as indicated by the relatively wide 95% LoA. Results from our relationship-scale approach corroborate data-scale findings, indicating moderately high accuracy of measures, but imprecision in the data, and thus only weak concordance correlation between reported and recorded latrine utilization measures.

As indicated in our methods, we assumed no gold standard measurement approach, thus our rationale for employing a type II modeling approach to carry out our comparative analyses. When selecting language to describe the results of our analyses, we had to choose one metric as a referent. We chose to use the more objective measure as the referent. As such, we describe respondent-reported use relative to sensor-recorded use and indicate that these data are overreported relative to sensor-recorded use. The reverse could also be said—that PLUM-recorded latrine use is under-estimated relative to respondent-reported use.

Although PLUM-recorded data represent more objective measures of latrine utilization compared with subjective reports, both metrics are susceptible to different types of bias. Given prior evidence of undercounting bias of sensor-recorded ‘likely defecation’ events in high-traffic scenarios,^16^ it is possible that the PLUM did not appropriately distinguish between ‘likely defecation’ events that occurred in immediate succession of each other. In addition to being prone to recall bias, respondent-reports may have been prone to courtesy bias, as defecating in a latrine reflects a behavior promoted by the WASH program that was under evaluation. As such, some respondents may have exaggerated latrine defecation events. However, socially-influenced factors (e.g., stigma surrounding the act of defecation [in any location, including a latrine], empirical and normative expectations regarding discussion thereof) may have caused some respondents to curtail reports of defecation events.

While reported latrine utilization is subject to reporting bias, when these measures are captured via the household use schedule, the data that are produced facilitate examination of more nuanced utilization trends within and across household settings. In other words, while respondent-reported use may be biased, it allows for individual-level assessments of latrine use patterns across age, sex and position within the household as well as household-level utilization assessments not possible with sensor-recorded data. These considerations are important when weighing options for latrine utilization measurement metrics, particularly for programs that intend to target utilization across late-adopting age and sex cohorts.

Our findings corroborate results from prior investigation that suggest levels of adoption of improved sanitation practices are at least moderately exaggerated.^22^ This is important, as programs and policies that are informed by reported data may not be on target to address sanitation uptake among different household members. These data also suggest it is necessary to obtain objective measures of latrine utilization, such as lower-cost sensors or latrine spot check indicators, to triangulate respondent-reported data and provide a more comprehensive assessment of latrine use. Employing sensors in a sub-set of program households can help determine whether and to what extent bias in reported latrine utilization exists, which may allow for adjustment and interpretation of respondent-reported utilization measures. Such bias may vary by population, setting, and cultural practices.

Our findings are consistent with results from several other studies that suggest respondent-reported data are susceptible to response bias. For example, our findings correspond with those of a recent study that found poor concordance between usual daily latrine use and average daily PLUM-recorded events and evidence of exaggerated reporting of household latrine use.^22^ That study found higher concordance between PLUM-recorded latrine use data and data captured on a 48 h recall as opposed to usual daily defecation and latrine use data. Similarly, a study of cookstove and water filter use also suggest overreporting of the utilization of these materials relative to sensor-recorded use.^11^ Other studies of respondent-reported handwashing indicate exaggeration of reported versus directly observed or instrument-monitored compliance as well.^4,6–10^ Unlike the handwashing literature, which indicates more severe overreporting of handwashing with soap relative to other more objective measures of the behavior,^9,23^ our results indicate relatively moderate bias in reported latrine utilization. The nature of the bias is indicated by the magnitude of the gradient of the mean measurement line relative to zero on the data-scale assessment, and the relative proximity of the reduced major axis to the line of perfect concordance on the relationship-scale assessment.

There are several limitations of this study. While the authors do not assume that day-to-day variation of latrine use events is uniform—thus the rationale for using language regarding ‘usual’ practices as opposed to ‘yesterday’s’ practices—inquiring about ‘usual’ practices does draw on an inherent assumption that there is some uniformity in practices. As a result, this underlying assumption may constrain, to some degree, the comparability of these respondent-reported and PLUM-recorded latrine use data. A relatively high proportion of defecation and latrine use data were obtained by having informants report on other household latrine users’ practices. These respondent reports may not be completely accurate. That being said, the use of proxies for the provision of reported defecation and latrine use measures is an efficient way of collecting data, and is a commonplace practice for assessing sanitation outcomes.^6^ In addition, our analytical sample was relatively small, with respondent-reported and PLUM-recorded data provided from 213 households, which may have contributed to the relatively wide limits of agreement. Furthermore, the cross-sectional study design prevented us from being able to assess temporal changes in agreement between measurement methods. Our data were captured during monsoon season and therefore may not represent usual defecation and latrine use practices carried out during other seasons of the year. To the greatest extent possible, PLUMs were installed on the same day enumerators obtained reported data. However, given time and resource constraints, we were unable to ensure contemporaneous collection of respondent-reported and PLUM-recorded data at all study households. This may not be an important limitation of this study, given the dearth of information regarding the impact of the timing of sensor placement and survey administration on the level of agreement between the two metrics. In some cases, it may even be preferable to avoid contemporaneous collection of reported and recorded latrine use measures. The algorithm used to detect defecation events was validated against direct observations of ‘likely defecation’ events. It is uncertain whether the algorithm detects child feces disposal, child latrine training, and menstrual hygiene management events as ‘likely defecation’ events, as the validation exercise did not explore these distinctions. However, respondent-reported utilization for defecation was higher than PLUM utilization rates, and one would expect that if these non-defecation-related sanitation events were included in PLUM-recorded measures they would serve to increase PLUM-recorded events and thus improve agreement between measures. Further research is needed to determine whether these latrine use practices are captured by the PLUM algorithm and whether respondents typically consider these events when responding to questions regarding latrine utilization. Irrespective of these limitations, our study findings provide important insights into assessments of latrine utilization and related measurement metrics.

## Conclusions

Given that respondent-reported latrine use is overreported relative to PLUM-recorded use measures, these data may not provide an accurate picture of sanitation program uptake. However, at the lower range of measurement values, overreporting is only modest, which indicates that reported data do not always immensely inflate sanitation utilization. This is worth considering, especially in contexts where latrine access is relatively high. Coupling reported utilization data with objective measures of use may further aid the estimation of latrine use. Program managers and policymakers should be mindful of moderate overreporting when using respondent-reported latrine use data to assess sanitation program progress.

In order to continue advancing the measurement of latrine utilization and defecation practices, and improving the evaluation of sanitation programming, the sector should continue pursuing several avenues. Further work is needed to transition from high-cost sensors used for research to simple, low-cost and acceptable sensors that can monitor latrine utilization amongst all household members, including the safe disposal of child feces in a sanitation facility. The availability of low-cost sensors could facilitate their incorporation into routine program monitoring, further increasing demand and lowering unit cost, thereby increasing opportunities for more objective measures of latrine use. Such sensors, or any other objective and reliable metrics of latrine use monitoring, could have the potential to transform donor funding to a pay-for-performance approach that reimburses implementing entities based on metrics of open defecation averted as opposed to the number of latrines built or the prevalence of latrine access. An approach such as this would further justify the cost of the sensors and their incorporation into sanitation programming. Similar work with sensors on handpumps and boreholes is currently under way.

As indicated above, PLUM signal data can be relatively imprecise, particularly in high-traffic scenarios. The sensors work well for monitoring household latrine utilization, but they do not work well for monitoring utilization at community, public or school facilities due to the limitations in the algorithm’s ability to distinguish between individual latrine events when there is a line of users. That being said, there may be an opportunity to investigate whether devices that take digitally blurred infrared photographs can be employed to distinguish between latrine use events while producing only anonymized ‘chalk drawing’ outlines.

## Supplementary data

Supplementary data are available at Transactions online (http://trstmh.oxfordjournals.org/).

## Supplementary Material

Supplementary Data
