# Supplementary material for: Comparison of respondent-reported and sensor-recorded latrine utilization measures in rural Bangladesh: a cross-sectional study
Source: Trans R Soc Trop Med Hyg. 2017 Nov 20;111(7):308–15. doi: 10.1093/trstmh/trx058 (PMC5914303; doi:10.1093/trstmh/trx058)
Supplement: Supplementary Data [file supplementalmaterial_revised_21sept17.docx]

**SUPPLEMENTAL MATERIAL**

**Comparison of respondent-reported and sensor-recorded latrine utilization measures in rural Bangladesh: A cross-sectional study**

Maryann G. Delea, Corey L. Nagel, Evan Thomas, Amal K. Halder, Nuhu Amin, Abul K. Shoab, Matthew C. Freeman, Leanne Unicomb, Thomas F. Clasen

**Table of contents**

- Detailed description of analytical methods
- Supplemental results
  - **Figure S1.** Distribution of four-day, household-level reported & recorded ‘likely defecation’ events
  - **Figure S2.** Distribution of residuals from regression modelling bias between methods
  - **Figure S3.** Pairplot of respondent-reported and PLUM-recorded ‘likely defecation’ events
- References cited in supplemental material

**Detailed description of analytical methods**

We used the following steps to generate relevant statistics and produce modified Bland-Altman and concordance plots:

1. *Calculation of relevant outcomes:* In accordance with the Bland-Altman method,^1^ and given the number of respondent-reported ‘likely defecation’ events for household *i* is $R_{i}$ , and the number of PLUM-recorded ‘likely defecation’ events is $P_{i}$, we calculated the difference between respondent-reported and PLUM-recorded utilization for a pair of household-level measures as $D_{i}=R_{i}-P_{i}$, and mean utilization as ${Av}_{i}={(R}_{i}+P_{i})/2$.^2^
2. *Testing of assumptions:* The Bland-Altman limits of agreement approach maintains two assumptions: 1) the distribution of differences in measures between methods follows an approximately normal distribution, and 2) the mean and standard deviation of the differences in measures are constant across the range of measurement.^1^ In order to test these assumptions, we:
   1. Examined the distribution of differences (i.e., residuals from a regression modelling bias between methods), and assessed the skew, kurtosis, and normal Q-Q plot to make a determination about the first assumption (Figure S2).
   2. Checked for non-constant bias and non-constant variance to make a determination about the second assumption. We modeled the mean bias between measurement methods, using linear regression, as $D_{i}=b_{0}+b_{1}\left( {Av}_{i} \right)$, where any “true” value of a latrine utilization measurement is estimated by ${Av}_{i}$.^2^ Non-constant bias was indicated by $b_{1} >0.$ We then regressed the absolute residuals ($B$) from the linear regression model on the average of the methods, as $B_{i}=c_{0}+c_{1}{(Av}_{i}).$^2^ Non-constant variance (i.e., heteroscedasticity) was indicated by $c_{1}>0.$The 95% limits of agreement were estimated as $b_{0}+b_{1}\left( {Av}_{i} \right) \pm2.46\{c_{0}+ c_{1}{(Av}_{i})\}$.^2^
3. *Generation of statistics related to the limits of agreement and modified Bland-Altman plot:* We modelled the direct relationship between the two measurement methodologies under comparison, using the *batplot* user-written package in Stata to do so. Given we detected non-constant variance and bias, we employed an extension of the Bland-Altman approach to produce a modified Bland-Altman plot.^2^ As a graphical display of the difference between and average of reported and recorded ‘likely defecation’ events, the modified Bland-Altman plot visualizes paired observations that fall within and outside of the 95% limits of agreement. The 95% LoA are represented by the dashed lines, and the mean is represented as a bold solid line in Figure 2, Panel A.
4. *Generation of CCC statistics and concordance plot, adjustment for skewed sampling distribution and underlying higher order effects:* We calculated the CCC for each household-level pair of latrine utilization measures.^3^ The CCC measures both precision (i.e., the deviation of observed measures from the line of best fit) and accuracy (i.e., the distance of the fitted line from the line of prefect concordance), and is therefore a more appropriate method for assessing agreement between measures from two methods of interest than the Pearson product-moment correlation coefficient, which only provides a measure of linear association between two variables, the intra-class correlation coefficient, and other regression-based comparative techniques.^1,4^ As a graphical display of observed concordance measures, the concordance graph plots observed measurement data from the two methods of interest, and includes the reduced major axis (i.e., bold solid line in Figure 2, Panel B) along with the line of perfect concordance (i.e., dashed line 45° from the x-axis in Figure 2, Panel B).^3^ The CCC increases in value as a function of the accuracy (i.e., the proximity of the data’s reduced major axis to the line of perfect concordance) and the precision (i.e., the tightness of the data about the reduced major axis) of the data. The reduced major axis serves as a summary of the center of the data, goes through the intersection of the means, and has a slope with a gradient provided by the sign of Pearson’s r and the ratio of the standard deviations. Unlike ordinary least-squares regression line, the reduced major axis is symmetric, which means that one line defines the bivariate relationship between the two variables of interest, regardless of which variable is considered the dependent variable.^5^ This is important for our comparative analyses, as we were not required to indicate one measurement method as the “gold standard”.

By definition, the concordance correlation coefficient is a scaled index of agreement that attains values numerically limited between -1 and 1. The -1 to 1 bounds on the concordance correlation coefficient therefore impart a skewness to the sampling distribution of the concordance correlation whenever the population correlation is not equal to zero.^6^ In order to prevent a skewed confidence interval, we employed a bootstrap re-sampling technique (1000 replications) to generate an accelerated bootstrap confidence interval ($\mathrm{BC}_{a}$) for the CCC presented herein.^7^

**Distinguishing between faulty and non-use PLUM data**

PLUM devices record the date and time of each motion detected by the passive infrared sensor with a resolution of three seconds. Once per day, at a time designated by the team (late at night), the PLUM logged the daily event data to a locally installed SD card. If no motion was detected within a 24-hour period, no data would be reported. However, a functional PLUM would still report a date and time upon each daily login. A faulty PLUM - one that was not recording any data - would not report at the designated time. In this way, we used the sensor data to distinguish between a non-use day accurately detected by a PLUM, and a faulty-PLUM wherein we did not consider the data for that given day in the analytical sample.

**Supplemental results**


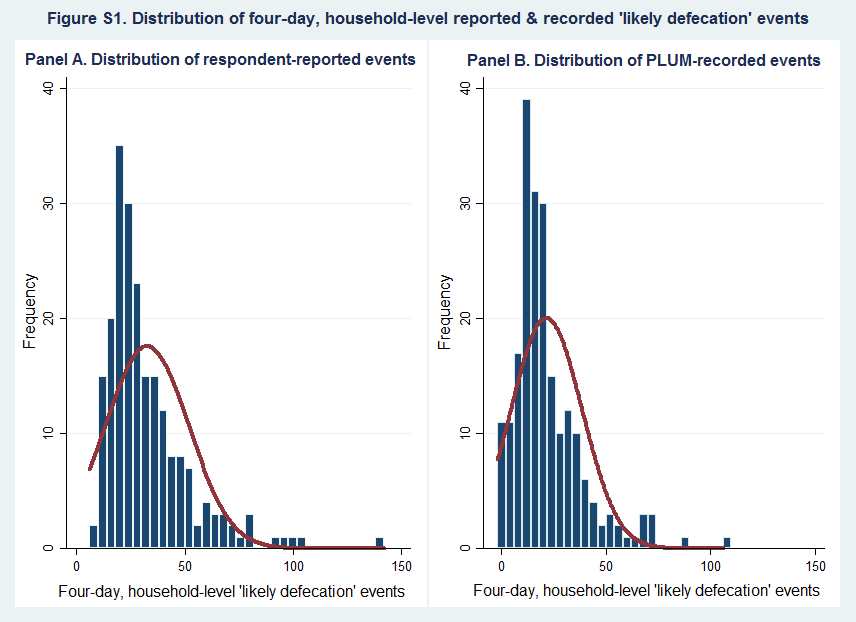
Utilization data generated by the two measurement methods were found to have similar distributions (Figure S1), meaning the data did not require further adjustment prior to conducting comparative analyses.

**Figure S1.** The histograms above indicate respondent-reported (Panel A) and PLUM-recorded (Panel B) outcome data had similar, over-dispersed, yet non-zero-inflated distributions, with extra variation compared to the mean.

**Results related to tests of assumptions**


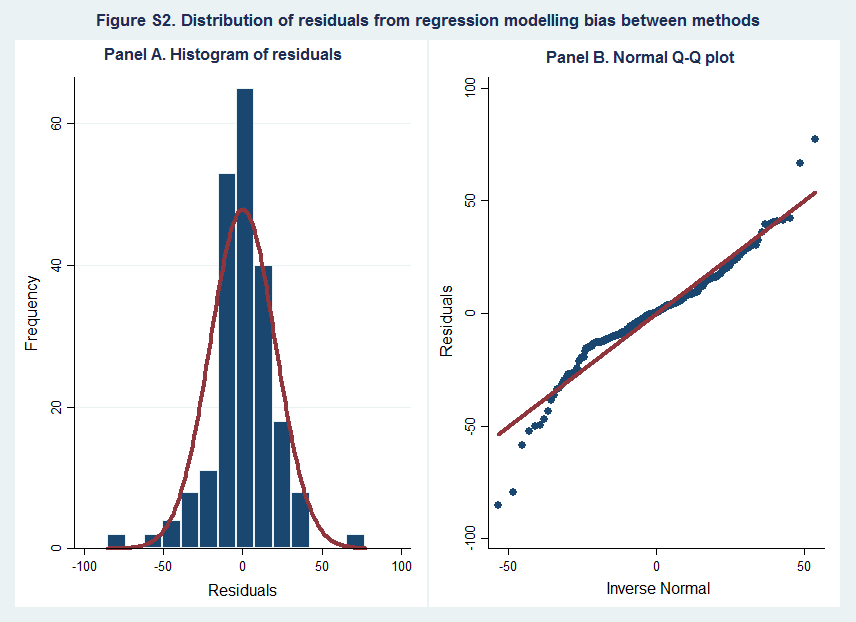
*Distribution of differences in measurements between methods.* An analysis of differences in household-level measurement pairs indicated that the distribution of differences between paired measures was not skewed, though it was leptokurtic, with heavy tails. The distribution of differences in ‘likely defecation’ events between methods was found to be approximately normal per histogram of residuals and normal Q-Q plot, so this assumption is valid (Figure S2).

**Figure S2.** The histogram (Panel A) and normal Q-Q plot (Panel B) reflecting the distribution of differences between household-level respondent-reported and PLUM-recorded latrine utilization measures indicate that the distribution was not skewed, though it was leptokuritic, with heavy tails. These findings indicate that one important assumption of the traditional Bland-Altman approach was not violated.

*Non-constant bias assessment*. We determined the bias was non-constant, as $b=0.19$, when we regressed the difference between the two methods on the average of the two methods. A pairplot of the difference versus average of respondent-reported and PLUM-recorded measures visualizes the structure of bias amongst all household-level measurement pairs, with the absolute difference in measures increasing as the average of the two methods increases (Figure S3). As a result, no simple statement can be made about the limits of agreement (i.e., the finite number of events over-reported relative to PLUM-recorded use) across the range of all utilization measurement values.


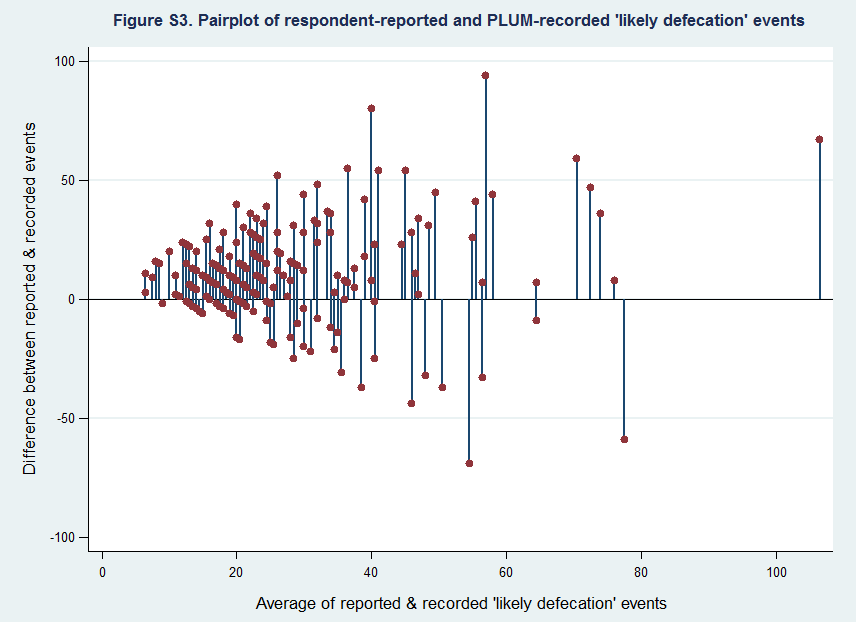


**Figure S3.** This pairplot illustrates that the bias in measurement methods increases (i.e., the spikes in the plot grow longer) over the range of average of respondent-reported and PLUM-recorded ‘likely defecation’ events. This pattern in the data indicates non-constant bias over the range of measures.

*Non-constant variance assessment.* We determined the variance was non-constant as well, as $\beta= 0.54$, when we regressed the absolute residuals predicted from the previous regression on the average of the two methods.

Since neither the bias nor the variance were constant over the range of utilization values (i.e., violating assumptions of the traditional Bland-Altman limits of agreement approach), it was necessary to employ a modified Bland-Altman approach that adjusted for non-constant variance and bias.

**References cited in supplemental material**

1. Altman DG, Bland JM. Measurement in medicine: the analysis of method comparison studies. *The Statistician* 1983; **32**(307-17).

2. Bland JM, Altman DG. Measuring agreement in method comparison studies. *Statistical methods in medical research* 1999; **8**(2): 135-60.

3. Lin LI. A Concordance Correlation Coefficient to Evaluate Reproducibility. *Biometrics* 1989; **45**(1): 255-68.

4. Zaki R, Bulgiba A, Ismail R, Ismail NA. Statistical Methods Used to Test for Agreement of Medical Instruments Measuring Continuous Variables in Method Comparison Studies. *PloS one* 2012; **7**(5): e37908.

5. Smith RJ. Use and Misuse of the Reduced Major Axis for Line-Fitting. *American Journal of Physical Anthropology* 2009; **140**: 476-86.

6. Cox NJ. Speaking Stata: Graphing agreement and disagreement. *The Stata Journal* 2004; **4**(3): 329-49.

7. Efron B. Better Bootstrap Confidence Intervals. *Journal of the American Statistical Association* 1987; **82**(397): 171-85.
